# Supplementary material for: The Role of Moral Distress on Physician Burnout during COVID-19
Source: Int J Environ Res Public Health. 2022 May 17;19(10):6066. doi: 10.3390/ijerph19106066 (PMC9142098; doi:10.3390/ijerph19106066)
Supplement: Supplementary file 1 [file ijerph-19-06066-s001.zip › ijerph-1667350-supplementary.pdf]

Table S1: Participant Demographics (N = 479)

Gender	f	%
Female	373	77.87%
Male	105	21.92%
They/Them	1	0.21%
**Age**	**f**	**%**
25-39	219	45.72%
40-55	228	47.60%
56-73	33	6.89%
**Region***	**f**	**%**
Northeast	117	24.42%
Southeast	71	14.82%
West	64	13.36%
Southwest	65	13.57%
Midwest	91	19.00%
Multiple regions	61	12.73%
Non-US	9	1.88%
Did not answer	1	0.21%
**Infected with COVID-19**	**f**	**%**
Yes	57	11.90%
No	421	87.89%
**Family infected with COVID-19**	**f**	**%**
Yes	184	38.41%
No	295	61.59%

*Participants identified the states in which they were licensed to practice. They were then coded using US Census standards into regions. When participants listed more than one state within the same region, they were counted as practicing within that region alone; when their area of practice straddled more than one region, they were placed into the "multiple regions" category.

Table S2: Means and Standard Deviations of Scales

Scale	Mean	Standard Deviation
OBI (burnout)	4.14	0.57
Perceived Organizational Support	2.90	0.97
Moral Distress	3.86	0.57
Workplace Motivation	1.88	0.44
Surface Emotional Labor	2.86	1.02
Deep Emotional Labor	3.52	0.68
Natural Emotional Labor	3.53	0.80
Problem-Based Coping	3.52	0.61
Emotion-Based Coping	3.32	0.43
